# Next-generation phenotyping: requirements and strategies for enhancing our understanding of genotype–phenotype relationships and its relevance to crop improvement

**DOI:** 10.1007/s00122-013-2066-0

**Published:** 2013-03-08

**Authors:** Joshua N. Cobb, Genevieve DeClerck, Anthony Greenberg, Randy Clark, Susan McCouch

**Affiliations:** 1 Department of Plant Breeding and Genetics, Cornell University, Ithaca, NY 14853 USA; 2 United States Department of Agriculture-Agricultural Research Service, Robert W. Holley Center for Agriculture and Health, Ithaca, NY 14853 USA; 3 Department of Biological Statistics and Computational Biology, Cornell University, Ithaca, NY 14853 USA; 4 Department of Biological and Environmental Engineering, Cornell University, Ithaca, NY 14853 USA

## Abstract

More accurate and precise phenotyping strategies are necessary to empower high-resolution linkage mapping and genome-wide association studies and for training genomic selection models in plant improvement. Within this framework, the objective of modern phenotyping is to increase the accuracy, precision and throughput of phenotypic estimation at all levels of biological organization while reducing costs and minimizing labor through automation, remote sensing, improved data integration and experimental design. Much like the efforts to optimize genotyping during the 1980s and 1990s, designing effective phenotyping initiatives today requires multi-faceted collaborations between biologists, computer scientists, statisticians and engineers. Robust phenotyping systems are needed to characterize the full suite of genetic factors that contribute to quantitative phenotypic variation across cells, organs and tissues, developmental stages, years, environments, species and research programs. Next-generation phenotyping generates significantly more data than previously and requires novel data management, access and storage systems, increased use of ontologies to facilitate data integration, and new statistical tools for enhancing experimental design and extracting biologically meaningful signal from environmental and experimental noise. To ensure relevance, the implementation of efficient and informative phenotyping experiments also requires familiarity with diverse germplasm resources, population structures, and target populations of environments. Today, phenotyping is quickly emerging as the major operational bottleneck limiting the power of genetic analysis and genomic prediction. The challenge for the next generation of quantitative geneticists and plant breeders is not only to understand the genetic basis of complex trait variation, but also to use that knowledge to efficiently synthesize twenty-first century crop varieties.

## Introduction

Agriculture faces tremendous challenges in the decades ahead. The FAO predicts that population and income growth will double the global demand for food by 2050, effectively increasing competition for crops as sources of bioenergy, fiber and for other industrial purposes (http://www.fao.org). Compounding the pressure for increased agricultural output are looming threats of water scarcity, soil fertility constraints, and climate change. Addressing these problems will require innovative approaches to both the agronomic and the genetic components of crop production systems. More sustainable management of renewable soil and water resources, in concert with more efficient utilization of genetic diversity will be key to achieving the necessary gains in productivity (Bakker et al. 2012; Frison et al. 2011; Cai et al. 2011; Pypers et al. 2011; McCouch et al. 2012).

Genetic diversity provides the basis for all plant improvement. Historically, plant breeders have sought to understand the nature of genetic variation by evaluating the performance of breeding populations over years and locations. Using replication and sophisticated experimental designs, they obtained useful insights about trait heritability, the influence of environment, the breeding value of different parents, and strategies for selecting genetically superior offspring in the field. With the dawn of the genomics era, emphasis began to shift toward the evaluation of genetic diversity directly at the DNA level. This approach is of interest to geneticists for the evolutionary and functional insights it brings, and to plant breeders as a source of tools for improving the power and efficiency of selection. Parallel investments in genotyping and phenotyping have generated datasets that can be associated with each other to address both basic and applied questions. Geneticists are interested in the nature and origin of mutations and their functional significance in the context of both qualitative and quantitative traits. Plant breeders embrace genomics as a way to document and protect the genetic composition of plant varieties, trace pedigree relationships, identify and select valuable mutations, and gain insight into the nature of genotype by genotype (G×G) and genotype by environment (G×E) interactions. The ultimate goal of genomics research in plant breeding is to contribute to improving the efficiency, effectiveness and economy of cultivar improvement.

As biology moves from a data-starved and largely observational discipline to a data-rich science capable of prediction, it follows the path of physics and engineering that came before. The tremendous innovation in genomics technology over the last two decades has been driven by multi-faceted collaborations between chemists, biologists and engineers, and today, costs continue to decline while accuracy and throughput continue to increase (Elshire et al. 2011; Tung et al. 2010). Correlated with the downward trend in the cost of sequencing is the expanded use of high-resolution genotyping in plant species that were previously ignored by the genomics community, a sampling of which include cassava, common bean, pea, sunflower, cowpea, and grain amaranth (Bachlava et al. 2012; Ferguson et al. 2011; Hyten et al. 2010; Maughan et al. 2011; Smýkal et al. 2012; Varshney et al. 2009; Varshney et al. 2010). In addition to offering new insights into diverse germplasm resources, high-throughput genotyping and next-generation sequencing (NGS) make it possible to efficiently leverage genetic information across species. The power of whole-genome sequencing as a unifying force in biology has motivated the development of diversity panels and large mapping populations in many crop species to facilitate trait dissection and gene discovery (Atwell et al. 2010; Huang et al. 2010; McCouch et al. 2012; Yu et al. 2008; Zhao et al. 2011a; Neumann et al. 2011; Pasam et al. 2012). It has also catalyzed new thinking about how to manipulate the genetic variation that exists in elite gene pools (Chen et al. 2011; Thomson et al. 2011; Trebbi et al. 2011).

With the deluge of low-cost genomic information on important crop species, a fundamental change in research emphasis is needed to address the shortage of high-quality phenotypic information. At this time, phenotyping has replaced genotyping as the major operational bottleneck and funding constraint of genetic analyses. Unlike genotyping, which is now highly mechanized and essentially uniform across organisms, phenotyping is still a cottage industry, species-specific, labor intensive, and inevitably environmentally sensitive. Further, while sequence variation is theoretically finite, and thus all sequence variants could conceivably be discovered for a given crop species, there is no expectation that the phenome will ever be fully characterized (Houle et al. 2010). The phenome of an organism is dynamic and conditional, representing a complex set of responses to a multi-dimensional set of endogenous and exogenous signals that are integrated over the evolutionary and developmental life history of an individual. Phenotypic information can be envisioned as a continuous stream of data that changes over the course of development of species, a population or an individual in response to different environmental conditions. While it can be associated with genomic information to understand the components of phenotypic variation that are due to genetics, with increasing availability of high-density genotypic information, understanding genotype–phenotype relationships is becoming more dependent on the availability of high-quality phenotypic and environmental information.

Over the next two decades, the development of phenotyping strategies will almost certainly mirror innovations in genotyping technology that have occurred over the last 20 years, characterized by increasing automation and throughput (Rafalski and Tingey 1993; Perlin et al. 1995; Sheffield et al. 1995; Weber and Broman 2001). As the science of phenotyping evolves, emphasis will increasingly be placed on generating information that is as accurate (able to effectively measure traits and/or performance characteristics), precise (small variance associated with replicated measurement), and as relevant as possible, while keeping costs within reasonable limits. If developments in genotyping offer a roadmap for where phenotyping is going in the future, these objectives will be reached based on new forms of automation and collaborations between biologists, engineers and computer scientists.

The purpose of this review is to outline considerations related to the future of phenotyping as the basis for association mapping and gene discovery as well as for developing predictive genomic selection (GS) models for crop improvement.

## Association between phenotype and genotype

The central challenge of modern genetic analysis is to understand the biological determinants of quantitative phenotypic variation. To date efforts in the plant genetics community have done well at identifying genes underlying traits controlled by one or a few loci with large effects. This is particularly true in the major crop species where genetic analyses have identified the biochemical basis of many important phenotypes (particularly, resistance to biotic and abiotic stress) and have also been the driving force behind the development of tools for marker-assisted selection in crop improvement (Foolad and Panthee 2012; Jin et al. 2010; Paux et al. 2010; Robbins et al. 2010). However, understanding complex trait variation has proven frustratingly difficult, as the genetic architecture of these important traits often involves many loci of small effect that may interact with each other as well as with the environment (Buckler et al. 2009; Collard and Mackill 2008; Schuster 2011). To discriminate such small effects, a combination of technologies and statistical methods are now being employed. NGS technologies have provided an economically feasible way to survey genetic variation with a resolution that is now limited more by the linkage disequilibrium (LD) in a particular mapping population than by marker density. This phenomenon has motivated the assembly of large panels of genetic diversity as well as the creation of large inter-mated populations to manipulate LD and facilitate the association of genotype with phenotype (Huang et al. 2011; Morris et al. 2013; Yu et al. 2008; Zhao et al. 2011a). These large and diverse populations aim to increase the recombination frequency and the frequency of rare alleles in order to enhance the power to infer the effects of individual loci. This also highlights the need for careful population design and advocates for the inclusion of admixed lines that may provide statistically useful observations of allele effects in diverse genetic backgrounds.

## Phenotyping for genomic selection

The emphasis on precision-phenotyping represents a significant change for breeders engaged in variety development who have traditionally favored simplicity, speed, and flexibility over sensitivity, precision and accuracy. This is because, historically the advantages of the latter could not be translated into economically relevant genetic gain in a breeding context. We argue that this paradigm is beginning to change with the potential to integrate GS into a variety development program. As the cost and efficiency of obtaining genomic information on large numbers of individuals dips below the cost and efficiency of evaluating populations phenotypically over years and environments, the breeding community is alert to the idea that genomic information can be leveraged to predict phenotypic outcomes (Cabrera-Bosquet et al. 2012; Heffner et al. 2009; Heslot et al. 2012). Further, the use of Bayesian models facilitates the analysis of sparse data (where not all individuals or families are evaluated phenotypically in each environment) and strongly suggests that there are cost-effective experimental designs that can dramatically reduce the amount of replication needed to extract meaningful phenotypic performance indicators for a population (see section on “Analysis, adjustment, and value extraction of phenotypic data”).

If the accuracy of genomic predictions is sufficient to offset the time and expense required to evaluate the performance of the breeding populations in the traditional manner, and if GS demonstrates a clear increase in the rate of genetic gain per cycle of selection, then breeders will quickly adopt the most efficient strategy to accomplish their goals. This may require staggered use of traditional and precision-phenotyping, depending on the trait(s) and the species under consideration. What is important is breeders begin to reevaluate how a focused investment in precision-phenotyping of a training population may be able to minimize the requirement for costly, extensive phenotyping of large numbers of lines every generation in the future. The purpose of this paper is to explore some of the key dimensions of next-generation phenotyping that will allow geneticists and breeders to productively interrogate the complex ménage-à-trois between genotype, phenotype and the environment as well as to develop models that leverage genotypic information to predict phenotypic outcomes.

Under a GS model, precision-phenotyping is most important when evaluating a training population because that dataset provides the basis for developing the statistical model that is then used to predict phenotypic performance in related members of a breeding population. The model is derived from the relationship between phenotype, genotype, and G×E, where marker genotypes are treated as random variables. GS is particularly useful when it can save a generation or two of time-consuming and expensive phenotyping, as only comparatively small training populations need be screened.

Genomic selection aims to model genome-wide SNP variation without concern for identifying particular alleles, loci or pathways or understanding how different alleles contribute to the phenotype. Since the metric of success is the ability to predict the performance of an adapted line or variety under relevant agronomic conditions, it is important to consider phenotyping strategies that (1) estimate crop performance under appropriate management conditions in the field; (2) can evaluate performance across a population of target environments; and (3) can generate useful data in real time without a disproportionate investment in labor and infrastructure. Despite the advantages of accelerating the breeding cycle, the ability of GS models to accurately predict phenotype is dependent on using prohibitively large training populations when working with traits with low heritability and complex inheritance (Calus et al. 2008; Guo et al. 2012; Hayes et al. 2009; Heffner et al. 2010; Jia and Jannink 2012; Kumar et al. 2012; Nakaya and Isobe 2012; Munoz et al. 2011; Resende et al. 2012; Zhao et al. 2011b; Zhong et al. 2009). This is due to the fact that G×E interaction plays a major role in explaining field performance, and GS is highly dependent on a prediction model developed from a limited sampling of the environmental variance. Recombination also disrupts phasing of markers and leads to low accuracy of predictions as breeding generations are farther and farther removed from the training population. Further research is needed to improve the accuracy of prediction under GS models.

## Phenotyping for QTL and gene discovery

In contrast to GS, phenotyping of a diversity panel for genome-wide association studies (GWAS) or a bi-parental mapping population for QTL analysis is designed to interpret and dissect the genetic architecture of complex traits and to understand how specific DNA variants condition the inheritance of diverse phenotypes. Both forms of linkage mapping are successful at implicating genomic regions involved in complex trait variation, but cloning the gene(s) underlying the QTL remains time-consuming and resource intensive, even when the QTL explains a substantial proportion of the phenotypic variation (Bhattacharyya 2010; Fan et al. 2006; Krattinger et al. 2009; Li et al. 2010; Liu et al. 2008; Saito et al. 2010). Bi-parental populations are limited by the particular alleles present in the parents, but they offer power for QTL dissection because population structure is disrupted and genetic background differences in the progeny are constrained. Association mapping studies, on the other hand, generally provide higher resolution of QTL for the same number of lines and evaluate a wider array of alleles but are limited by the inability to interrogate rare alleles or to dissect phenotypes that are perfectly correlated with population structure (Manolio and Collins 2009; Price et al. 2006; Pritchard and Cox 2002; Reich and Lander 2001). When large numbers of markers are used for either QTL analysis or GWAS, a multiple test correction is required to limit the false discovery rate. With ever-improving approaches to statistical modeling and improvements in the accuracy and precision of phenotyping, both forms of linkage mapping hold great promise for elucidating the genetic architecture of complex traits and identifying the genes and specific alleles underlying trait variation.

## Sampling vs. controlling environmental variation

Different approaches to phenotyping are required for different purposes (Campos et al. 2004; Crouch et al. 2009; Gordon and Finch 2005; Kloth et al. 2012; Masuka et al. 2012; Pieruschka and Poorter 2012). Plant breeders have traditionally relied on large-scale replication of phenotypic trials over years and locations to identify individual families or populations that perform best in a target population of environments (TPE). By modeling locations and years as random effects, they were able to reliably extract genetic signal from environmental noise and identify varieties with broad or narrow zones of adaptation (Beavis 1998), though the process was very time and labor consuming. Many geneticists, on the other hand study phenotypic variation at the cell or tissue-specific level using plants grown under carefully defined environmental conditions, and evaluate cascades of molecular events using biochemical and “omics” technology. The world of the plant breeder and that of the molecular geneticist intersect at the level of the plant, but the different scales of phenotyping make it challenging to integrate the knowledge contributed by each community into a unified and comprehensive view of the genetic determinants of plant growth, development and response to environment.

Under field conditions, it is often convenient to collapse quantitative phenotypes into discrete categories to facilitate manual data collection in real time and at reasonable cost. This has been the practice for many years among breeders and geneticists working with large, field-grown populations, and different communities of researchers have developed standardized categorical scales or indices for important whole-plant phenotypes that are easy to apply (Clarke et al. 1992; De Boever et al. 1993; International Rice Research Institute 1996; Kuhn and Smith 1977; Molina-Cano 1987; Yuan et al. 2004). For example, traits such as flowering time or disease resistance are frequently estimated using a visual assessment of “days to 50 % flowering” in a row or plot, or “percent leaf area affected” on individual diseased plants. Historically, trait evaluation using these indices was reliable enough to provide reasonable data in the context of plant breeding. However, new population designs (Yu et al. 2008) in combination with high-density marker coverage have increased the power to detect small-effect QTL and estimate their effects, even on whole-plant phenotypes. This suggests that more rigorous, quantitative approaches to phenotyping are likely to bring rewards. Further, when there is significant variability in phenotypic scores collected by different individuals, more objective phenotyping protocols are desirable (Poland and Nelson 2010).

Recently, it has been argued that automated, high-throughput, field-based organismal phenotyping techniques involving remote sensing (such as near-infrared spectroscopy mounted on agricultural harvesters to measure spectral canopy reflectance with the aid of global positioning system (GPS)-guided tractors) will enhance the precision and accuracy of phenotyping without extracting plants from the production environment (Cabrera-Bosquet et al. 2012; Houle et al. 2010; Montes et al. 2007; Tuberosa 2012; White et al. 2012). While these efforts can certainly facilitate selection for enhanced performance in a target zone of adaptation, one of the biggest challenges associated with these automated, field-based technologies is the variable nature of most natural environments.

To enhance the ability to screen for stress tolerance in field-grown plants, scientists often use plant populations to ‘sample’ the degree of stress encountered in a TPE. Once this has been ascertained, the TPE is used to evaluate the relative performance of different populations over several growing seasons. This requires significant up-front investment, as many different locations must be tested over multiple years in order to make an accurate estimation. Alternatively, breeders use “managed stress” as a way of optimizing screening protocols for application to large plant populations in the field. By managing the amount and timing of water, fertilizer, pest control or soil amendments, plants can be exposed to fairly reliable levels of stress while experiencing normal temperature, day length, etc., over the course of the growing season. These approaches work well if the genetic component of phenotypic variation (heritability) is high, and if the differences among populations or individuals within a population are large. However, in cases where complex traits are conditioned by many alleles with small effects, the error associated with estimating the phenotype and the environmental variance contributing to the observed phenotypic variation are likely to dilute the relatively weak genetic signals and may preclude their detection.

To partially overcome this problem, many researchers have endeavored to take advantage of phenotyping strategies based on analytical chemistry (i.e. gas chromatography–mass spectroscopy, high performance liquid chromatography, inductively coupled plasma spectroscopy, etc.) or a wide range of -omics technologies (transcriptomics, metabolomics, ionomics, proteomics, etc.). These are all highly automated and are important and useful due to their high throughput and high accuracy. They are generally used to analyze specific anatomical parts of a plant at a particular time(s) in its development, and are best used on plants grown under well-defined growing conditions. Owing to the high cost per sample and the requirement for considerable technical expertise and infrastructure, these techniques may not be available to everyone and it may not be economically feasible to survey large numbers of field-grown plants. Thus, it often makes sense to first screen a population under controlled conditions with minimal replication and once a hypothesis about the genetic control of a trait of interest is formulated, it can be tested in a focused way in the field, or simply used to eliminate a large proportion of a population prior to undertaking field evaluation.

Screening populations under controlled conditions is also appropriate when the controlled environment is necessary to impose a particular form of stress or to permit growth of plants under specific conditions that cannot be replicated in the field. Controlled environments have been successfully used to inoculate plants with a particular strain of a pathogen, or to impose a particular abiotic stress such as aluminum toxicity without the natural coupling with phosphorus deficiency, or high CO_2_ in combination with a critical night time temperature. Use of multi-step strategies involving both controlled and field environments are often the best way to maximize the extraction of useful genetic information while minimizing the expense and time involved (Fernie and Schauer 2009; Rafalski 2010).

## Drought tolerance as an illustration

While a complete survey of advances in drought phenotyping is beyond the scope of this review (see Mir et al. 2012 for a detailed overview of this topic), drought tolerance offers a compelling example of a combined approach of leveraging both controlled and uncontrolled phenotyping designs to enhance genetic analysis. The onset of water deficit and its impact on plant performance is a dynamic process that occurs across space and time. Under field conditions the inability to obtain standardized and consistent drought stress contributes to a loss in heritability and presents a challenge for both selection and mapping experiments (Berger et al. 2010). Many different approaches have been used to apply defined levels of drought stress in an effort to understand the nature of this complex trait, ranging from chemically manipulating osmotic balance in hydroponics (Rengasamy 2010; Tavakkoli et al. 2010) to conveyer systems in glasshouses with digitally controlled irrigation systems (Granier et al. 2006; Jansen et al. 2009; Neumann 2013; Pereyra-Irujo et al. 2012) to the use of rainout shelters in the field (Czyczyło-Mysza et al. 2011; Dodig et al. 2012; Zhu et al. 2011a). Measurements of drought tolerance likewise range from surveys of root system architecture (Ibrahim et al. 2012; Landi et al. 2010; Lopes et al. 2011; Steele et al. 2007; Zhu et al. 2011b; Clark et al. 2011) to physiological metrics related to water status (Bartlett et al. 2012a, b; Blum 2009; Gilbert et al. 2011; Ogburn and Edwards 2012; Tucker et al. 2011) to spectral imaging of shoot tissue (Berger et al. 2010; Goltsev et al. 2012; Liu et al. 2011; Zia et al. 2012) to simply evaluating yield under stress in the field (Bennett et al. 2012; Bernier et al. 2007; Ghimire et al. 2012; Golabadi et al. 2011; Messmer et al. 2009; Rehman et al. 2011; Swamy et al. 2011; Venuprasad et al. 2012; Vikram et al. 2011). Screening can be done using in-house facilities (growth chambers, green houses) or outsourced to a phenotyping facility such as the Jülich Plant Phenotyping Centre—JPPC (Jülich, Germany; http://www.fz-juelich.de/ibg/ibg-2/EN/About_us/organisation/JPPC/JPPC_node.html9), the Leibniz-Institut für Pflanzengenetik und Kulturpflanzenforschung—IPK (Gatersleben, Germany; http://www.ipk-gatersleben.de/), the Plant Accelerator^®^ (University of Adelaide, Australia; http://www.plantaccelerator.org.au/), or the High Resolution Plant Phenomics Center—HRPPC (CSIRO Plant Industry, Canberra Australia; http://www.plantphenomics.org/HRPPC). The two former facilities are part of the larger European Plant Phenomics Network (http://www.plant-phenotyping-network.eu/) and the latter two are part of the Australian Plant Phenomics Facility (http://www.plantphenomics.org.au/). Each system presents its own advantages and disadvantages, but collectively they empower the researcher to investigate plant response to drought in ways that are more comprehensive than any one design can offer. These approaches are most often utilized for linkage mapping and gene discovery, and once QTL or candidate genes are identified, they can be validated for practical application by evaluating specific germplasm, genetic stocks or breeding populations under managed drought conditions in the field (Ali et al. 2010; Cavanagh et al. 2008; Huang et al. 2012; Kholová et al. 2010; Saisho and Takeda 2011; Venuprasad et al. 2011; Yadav et al. 2011). If validated, lines carrying the genes or QTLs of interest will be useful for elucidating the molecular mechanism(s) involved in the component trait(s), and will also be of immediate value as donor material for breeding with elite germplasm.

**Fig. 1:**
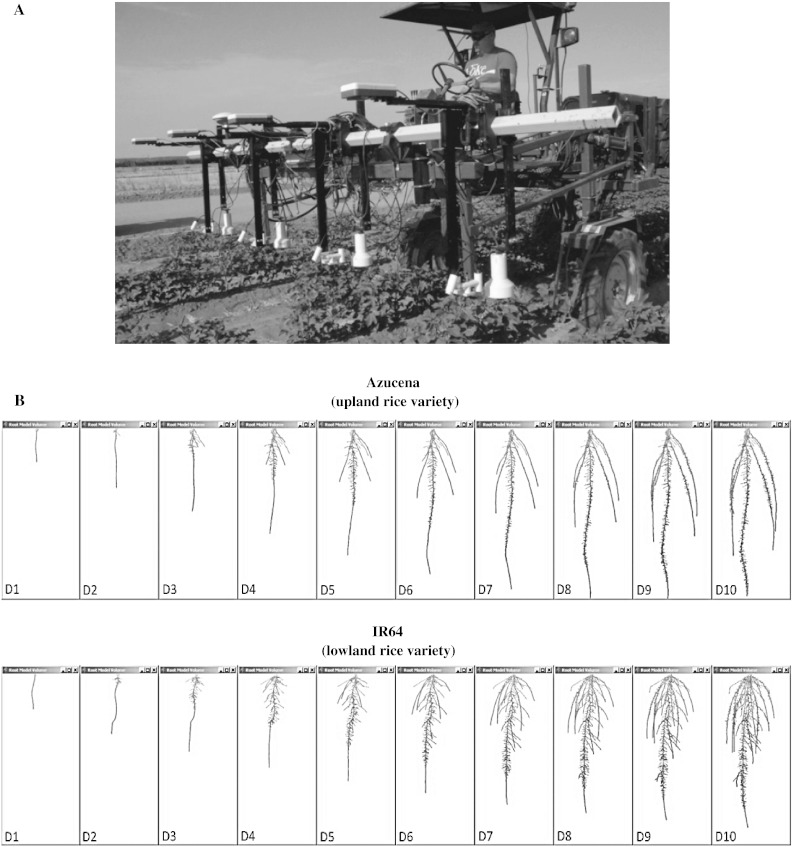
The choice of phenotyping under controlled conditions vs. field environments depends greatly on the purpose of phenotyping, heritability of the trait, and logistical considerations of data collection. **a** High-clearance tractor measuring the height, temperature, and spectral reflectance of young cotton plants. Such a system is reasonably high-throughput and can measure canopy traits with high accuracy and precision. These traits typically have high heritability and are considered component phenotypes of yield under drought stress (reprinted from White et al. 2012, copyright 2012, with permission from Elsevier). **b** Ten-day time course of root system growth in three dimensions of two divergent varieties of rice from Clark et al. (2012). Roots are notoriously difficult to phenotype in the field and root architecture in particular. This phenotype lends itself well to controlled conditions as the logistics of evaluating roots are more tractable, and it permits the exploration of otherwise un-surveyable phenotypes such as center of mass and dynamic tracking of architecture development over time (copyright American Society of Plant Biologists)

Examining the relationship between phenotypic variation under controlled environments and that observed under field conditions offers valuable insights that can be used to iteratively improve controlled environment phenotyping techniques so they are more predictive of plant performance in the field (Table 1; Fig. 1). Ultimately, the choice of phenotyping approach will depend on the intention of the researcher, the size of the population in question [e.g. less than ten individuals for precise physiological experiments, to a moderate number of lines (200–400) for mapping studies or GS training populations, or a large number of lines (400–1,000+) for association studies], the heritability of the phenotype, the tractability of the phenotype to controlled environment testing, and resource availability.

**Table 1:** Factors to consider when evaluating if a field-based or environmentally controlled phenotyping platform is most appropriate

Controlled conditions	Field conditions
Minimizes environmental variation and increases heritability	Maximizes relevance to breeders and farmers
Increased precision of critical measurements	Characterizes the range of environmental variation
Maximizes information from a minimum of replicates	Evaluates over time as well as space
Decreases cost through automation and standardization	Estimates genotype × environment interaction
Develop hypotheses to be tested on a targeted set of lines in the field	Refine hypothesis and develop new screening protocols

## Development of technology and phenotyping tools

The creative use of technology and careful development of tools to automate processes without sacrificing predictive power will be critical as next-generation phenotyping platforms are developed. This can be a real challenge as many experimental techniques in plant physiology, molecular biology and breeding can be restrictive and require specialized protocols that are often difficult to standardize. The integration of these approaches will be necessary to fully interrogate the genetic landscape of complex traits. Standardized phenotyping systems are not feasible for all research questions, but with thorough consideration and clearly defined objectives, many techniques can be harnessed to investigate specific traits under high-throughput settings.

In recent years, automation, imaging, and software solutions have paved the way for many high-throughput phenotyping studies. Semi-automated systems have been successfully applied to investigate various components of plant growth and development, and can be used to help tackle basic research questions when combined with genetic mapping strategies (Famoso et al. 2010). Additionally, automated systems have allowed researchers to reduce the labor needed to manage and perform large-scale growth screens in laboratory, greenhouse and field environments (Nagel et al. 2012).

Aside from mechanization, digital imaging has emerged as a cornerstone to capturing quantitative phenotypic information under most automated or semi-automated approaches. Imaging has allowed many aspects of plant development, function, and health to be monitored, measured and tracked in ways previously unattainable using conventional metrics. Large image data sets, however, require novel software solutions in order to process and extract meaningful estimates of phenotypic variation. Most image analysis tools for plant phenotyping incorporate predefined processing and analysis procedures into semi-automatic or automatic routines in order to quantify multiple phenotypes from single images or groups of images.

In its essence, high-throughput phenotyping means integrating and optimizing a phenotyping process in a way that makes it as efficient and controlled as possible. In considering efficiency, several questions and decisions arise related to the accuracy, precision, automation, and adaptability of various stages of the phenotyping process, from growth techniques to experimental design and management practices to data capture and analysis strategies.

The accuracy and precision of the treatment and measurement process is a fundamental concern during any experimental procedure. During phenotyping studies where multiple individuals and replicates from different genetic backgrounds are evaluated across batches, effectively controlling the accuracy and precision of the phenotyping system will have direct impacts on the outcome of the analysis. Accuracy and precision are intimately interrelated, where accuracy represents how close the process or measurement is to the absolute truth and precision represents the repeatability or variance of the measurement process. Accuracy is important when there is variation across individual genotypes during mapping experiments. For instance, Clark et al. (2011) characterized the root systems of a rice bi-parental recombinant inbred line (RIL) population, and found that one parental genotype had dense, highly branched root systems while the other had long, sparse root systems. In order to clearly capture these differences, a system needed to be designed that could correctly quantify both types of root systems in order to properly assess their relationship and further analyze variation within the progeny. Comparison and validation studies with known standards, such as the use of complementary imaging modalities or other quantification software, can help evaluate the accuracy of a system.

Precision is critical when individual genotypes have multiple replicates that are evaluated across several batches. Presuming that the replicates share similar characteristics, the measurement system must be able to quantify the features in a repeatable way to prevent unpredictable system noise from masking the true similarities/differences between the genotypes. While there are statistical approaches for accounting for unwarranted variability in silico, efforts to improve the precision of data collection will only serve to enhance the statistical power of any analysis performed. The key to maintaining precision throughout a phenotyping activity is to employ stable instrument designs that can effectively control precision, such as the fixed lighting and camera setups used in the root systems in studies cited previously.

Unfortunately, there will always be trade-offs between the maintenance of accuracy, precision, and the ultimate throughput of the phenotyping approach. As throughput and standardization increase, it necessitates a drop in accuracy and precision that must be carefully monitored in order to maintain the economic feasibility of the data collection. It is not always straightforward to properly balance these trade-offs, but through iterative design and testing, phenotyping tools can be established to satisfy research objectives and meet resource constraints.

The level of automation employed by a phenotyping approach is counter-balanced not only by trade-offs with accuracy and precision, but also with adaptability. Increasing automation improves throughput and reduces labor costs, but also results in more specialized designs that have less adaptability and predictive power, and are prone to errors from non-standard individuals. This principle is illustrated well when image analysis involves batch processing many photographs using predefined algorithms and commands. It is fairly obvious that batch processing is invaluable during large-scale phenotyping experiments where thousands of images can be generated daily, but this also means that the software must rely on a rigid set of constraints. The quality of the images is usually not a problem during high-throughput phenotyping where the imaging process is standardized, but if any individuals deviate from pre-specified growth assumptions of the measurement algorithm, unpredictable and misleading measurement errors can arise. Even with automated analysis algorithms that have been thoroughly tested, it is necessary for the experimenter to manually check and validate the system outputs regularly. Along those same lines, incorporating user-guided processes into the phenotyping pipeline can also provide a useful compromise that improves the flexibility while maintaining the efficiency needed to perform large experiments (Clark et al. 2012; Le Bot et al. 2010; Lobet et al. 2011).

Most phenotyping tools that have been developed by research groups in the public and private sector are integrated in a way that makes them easy to disseminate and use, but sometimes this convenience can limit the range of their functionality to other studies. While this has precipitated the release of a number of software programs available for the extraction of phenotype data from images (Table 2), the highly specific nature of individual phenotypes also motivates the development of in-house tools ideally suited to the analysis at hand. Although it is not a simple task, implementing modular designs will help increase flexibility of phenotyping in the future. The ImageJ analysis tool is a good example of the successful incorporation of modular designs in the software realm (Schneider et al. 2012). This image processing software allows users to create and share custom-developed plugins that expand the functionality of the software and make it applicable to wide range of research disciplines. Modular concepts have proven quite successful for the high-throughput phenotyping of notoriously difficult phenotypes such as root system traits. A notable example is GiaRoots, a software program that allows users to incorporate their own processing and algorithms into the automated analysis routines as a way of overcoming the limitations imposed by more integrated approaches (Galkovskyi et al. 2012).

**Table 2:** Selected image analysis software programs and phenotyping platforms available for high-throughput phenotyping

Tissue	Software	Purpose and design	Reference
Roots	WinRhizo Tron	Morphological descriptions of root area, volume, length, etc	http://www.regent.qc.ca/products/rhizo/RHIZOTron.html
	KineRoot	2D analysis of root growth and curvature	Basu et al. (2007)
	PlaRoM	Platform for measuring root extension and growth traits	Yazdanbakhsh and Fisahn (2009)
	EZ-Rhizo	2D analysis of root system architecture	Armengaud et al. (2009)
	RootTrace	Counting and measuring root morphology	Naeem et al. (2011), French et al. (2009)
	DART	2D analysis of root system architecture	Le Bot et al. (2010)
	SmartRoot	ImageJ plugin for the quantification of growth and architecture	Lobet et al. (2011)
	RootReader3D	3D analysis of root system architecture	Clark et al. (2011)
	RootReader2D	2D analysis of root system architecture	Clark et al. (2012)
	Gia-Roots	2D analysis of root system architecture	Galkovskyi et al. (2012)
Shoots/leaves	WinFolia	Morphological measurements of broad leaves	http://www.regent.qc.ca/products/folia/WinFOLIA.html
	TraitMill	Platform for measuring various agronomic characteristics	Reuzeau et al. (2006)
	PHENOPSIS	Automated measurement of water deficit-related traits	Granier et al. (2006)
	LeafAnalyser	Rapid analysis of leaf shape variation	Weight et al. (2007)
	LAMINA	Quantification of leaf size and shape	Bylesjö et al. (2008)
	HYPOTrace	Analysis if hypocotyl growth and shape	Wang et al. (2009)
	LEAFPROCESSOR	Analysis of leaf shape	Backhaus et al. (2010)
	Lamina2Shape	Analysis of lamina shape	Dornbusch and Andrieu (2010)
	HTPheno	ImageJ plugin for morphological shoot measurements	Hartmann et al. (2011)
	LEAF-GUI	Analysis of leaf vein structure	Price et al. (2011)
	LemnaTec 3D Scanalyzer	Comprehensive platform for analysis of color, shape, size, and architecture	Golzarian et al. (2011), http://www.lemnatec.com/
Seeds/grain	WinSEEDLE	Volume and surface area measurements of seeds and needles	http://www.regent.qc.ca/products/needle/WinSEEDLE.html
	SHAPE	Quantitative evaluation of shape parameters	Iwata and Ukai (2002), Iwata et al. (2010)
	ImageJ	General image analysis software for area, size, and shape; applied to grain	Herridge et al. (2011), http://rsb.info.nih.gov/ij/
	GROWSCREEN-Rhizo	Simultaneous analysis of growth rate, leaf area, and root growth	Nagel et al. (2012)
	SmartGrain	High-throughput measurement of seed shape	Tanabata et al. (2012)

## Access, storage, and management of phenotypic data

It is clear then, that much of next-generation phenotyping will be done at the intersection of the fields of biology, engineering, and computer science. Progress in developing technology in these disciplines that empowers next-generation phenotyping strategies is moving forward rapidly. However, congruent with the progress in the capability to collect high-throughput phenotypic data, is the growing problem of managing these data sets in ways that empower value extraction. Retrofitting a lab to handle the rapid influx of phenotype data could require significant investment in facilities, device control systems and computational resources.

For experiments that are only measuring a few traits on a panel of germplasm, setting up a local (customized) phenotyping system in-house might be practical; but in such cases, a laboratory information management system (LIMS) or local database may be needed to manage the high volumes of phenotypic information. Generally, there are no ‘off the shelf’ solutions that can be applied universally, so some computer expertise will be needed for data management. Even so, organizing that information into a “phenome” is challenging because of the continuous, multi-faceted, and interpretive nature of what a phenotypic observation is, contrasted with the “discrete” nature of genotypic data, which can be abstracted into a single alphabetical character (National Science Foundation 2011).

Beyond the technologies used to run, collect and digest large-scale phenotypic evaluations, the field of phenomics faces similar bottlenecks that genomics has been grappling with as the drop in cost of DNA sequencing outpaces the cost of hard drive data storage (Stein 2010). Though there is ample exploration that can be done on genomic data alone, for many plant researchers, associating and enriching genotype data with phenotypic manifestations contextualized by the field environment is a vital part of gaining true biological insight and solving agronomic problems.

The storage of phenotype data at this scale has become a sub-discipline on its own and some projects are dealing with it quite well. There are many public databases that have been working to organize and collate plant phenotype data (Lai et al. 2012; Table 3), but most only have the current capacity to present free-text phenotypic descriptions of mutants, e.g. SoyBase (Grant et al. 2010) and MaizeGDB (Schaeffer et al. 2011). Some crop databases have tried to move beyond this paradigm by including functionality for the management of phenotypic measurements, predominantly from either managed field trials or GWA studies [e.g. T3 Triticeae Toolbox (http://triticeaetoolbox.org), Panzea (Canaran et al. 2008), and Gramene’s diversity module (Chen et al. 2010)]. They are also among a number of projects preparing for an increasing amount of association data emerging from the marriage of powerful genomic information with next-generation phenotyping. One effort is NCBI’s dbGAP (Mailman et al. 2007), which was created as a public repository for phenotypes, genotypes and the associations between them. Currently, however, dbGAP only accepts human data.

**Table 3:** A selection of integrated storage and maintenance systems designed to enable query-based approaches to characterizing variation in phenotypic data and the relationship it shares with genotypic data

Taxa	Database	Organism(s)/taxa	Website	References
Plant	MaizeGDB	Maize	http://www.maizegdb.org/	Schaeffer et al. (2011)
	Panzea	Maize; Teosinte	http://www.panzea.org/	Canaran et al. (2008)
	PHENOPSIS DB	*Arabidopsis*	http://bioweb.supagro.inra.fr/phenopsis/	Juliette et al. (2011)
	Gramene Diversity Module	*Arabidopsis*; rice; maize; sorghum; wheat	http://www.gramene.org/db/diversity/diversity_view	Chen et al. (2010)
	IonomicsHub	Arabidopsis; rice; yeast; soybean; maize	http://www.ionomicshub.org/home/PiiMS	Baxter et al. (2007)
	Oryza Tag Line (OTL)	Rice	http://oryzatagline.cirad.fr/	Larmande et al. (2008)
	Rice Mutant database (RMD)	Rice	http://rmd.ncpgr.cn/	Zhang et al. (2006)
	T3 Triticeae Toolbox	Wheat; barley	http://triticeaetoolbox.org/	Blake et al. (2012)
	Tomato Mutant Database	Tomato	http://zamir.sgn.cornell.edu/mutants/	Menda et al. (2004)
	SGN	Solanaceae species	http://solgenomics.net/	Bombarely et al. (2011)
Non-plant	PhenomicDB	Human; mouse; fruit fly; yeast; zebrafish; slimemold; nematode	http://www.phenomicdb.de/	Kahraman et al. (2005)
	MGI	Mouse	http://www.informatics.jax.org/	Eppig et al. (2012)
	The Phenoscape project	Numerous	http://kb.phenoscape.org	Mabee et al. (2012)
	dbGAP	Human	http://www.ncbi.nlm.nih.gov/gap	Mailman et al. (2007)

There are a few database projects that specialize specifically in plant phenomics data, and deserve to be highlighted. The first example of these is PHENOPSIS DB (Juliette et al. 2011), which mainly houses information regarding the growth response of *Arabidopsis thaliana* to various environmental conditions. The database is populated with phenotype information extracted from images and measurements collected automatically in specialized growth chambers. The collaborative international network for ionomics ( http://www.ionomicshub.org; Baxter et al. 2007) is a second example that hosts ICP-mass spectrometry ionomics data for thousands of *Arabidopsis*, rice, and yeast samples with the goal to facilitate the understanding of response mechanisms in plants to various nutrient availabilities and/or abiotic toxicities.

Additionally, there are other efforts in human and mouse genomics research that could serve as useful models for continued development in the plant phenomics domain. Mouse genomics informatics (MGI), (http://www.informatics.jax.org) comprise several database projects, including the mouse genome database (MGD), (Eppig et al. 2012) and houses a variety of tools for searching and browsing large phenotype data sets. PhenomicDB is another, multi-species (primarily human, mouse, fruit fly, and yeast) resource designed to empower “comparative phenomics” (Kahraman et al. 2005). The nutritional phenotype database (Van Ommen et al. 2010) is a third, which focuses on human nutritional phenotype data. The DbNP even goes a step further than most databases by emphasizing the importance of the characterization and unification of experimental designs and allows for finer grained storage and searching protocol parameters. The DbNP project recently announced that it will further expand the scope of the resource to include management of environmental plant studies (http://www.dbnp.org).

One important feature shared among many current databases organizing phenotype data is the use of controlled vocabularies known as ‘ontological terms’. Ontologies are sets of defined keywords that can be used as tags to qualify and describe features related to biological data points and data sets. Such ontological terms can be used to describe traits, genes, environments, or taxonomy. As an example, one might use the hierarchy of terms “growth and development ≥ shoot development ≥ inflorescence development ≥ flower development ≥ flowering time ≥ days to flower” to describe formally what is colloquially referred to as simply “flowering time”. While this is an arguably simple example, it is not difficult to imagine the complexity that ensues when trying to use ontologies to describe complicated molecular pathways. Usage of ontologies is a critical step toward making diverse and rapidly growing collections of biological data searchable, and accessible to computational algorithms. The Open Biological and Biomedical Ontologies Foundry (OBO foundry) (Smith et al. 2007) has emerged as an important centralized repository for plant and animal ontological collections, with the goal of increasing standardization and maximizing interoperability between ontologies. For plant data the most commonly used ontologies include the plant ontology (PO), (Avraham et al. 2008; Jaiswal et al. 2005) the plant trait ontology (TO), plant environment ontology (EO), and the phenotypic quality ontology (PATO).

Unfortunately, all research groups do not universally adopt usage of these community standards and without a critical mass of “buy-in”, their benefits cannot be fully realized. Also a great deal of time and resources go into the curation and maintenance of ontologies and projects rely on term-based grant funding, which is not always reliable.

In order to meet the demand imposed by the upscaling of phenotypic data production, sophisticated computational methods will need to be employed. Phenotype data is complex and highly context sensitive, and crucial information can potentially be lost when data descriptions are flattened down to only a few ontology terms. As a way of dealing with this complexity, some groups have been exploring the potential of the Semantic Web (Lee et al. 2001) to expand the dimensionality of stored biological data in order to more effectively mine the enormous volume of descriptive data available in the literature (Vision et al. 2011). The Phenoscape project (http://kb.phenoscape.org; Mabee et al. 2012) has been working on developing semantical search algorithms capable of linking biological data by relationships between ontological terms and by similarities found between free-text descriptions. Data are characterized as statements of fact, where there is a subject (e.g. “a floret”), a predicate (e.g. “has the color”) and an object (e.g. “white”). Capturing phenotypic metadata using this approach adds some of the necessary dimensionality for unlocking biological meaning using linguistic and intuitive tool sets.

## Analysis, adjustment, and value extraction of phenotypic data

Independent of the effort involved to both collect and appropriately manage high-throughput phenotype data, the data sets themselves are only as valuable as the analyses that can be performed on them. Great care must be taken to make accurate inferences from the data in order to correctly characterize the genotype–phenotype relationship. Correct estimations of genetic gain from selection, for example, depend heavily on accurate estimates of heritability and the covariance among phenotypes (Dickerson 1955). Because none of these parameters are directly observable, they must be estimated from data using a variety of statistical models.

While the methods for measuring phenotypic data are becoming more sophisticated and the ability to catalog and query data across experimental designs is becoming more achievable, the precision of such data will always be limited by inherent biological noise. This biological noise is unavoidable and is even present under the most controlled experimental conditions. These fluctuations can be local, affecting single organisms, or more general, influencing the whole experiment and modifying the phenotypes for the whole replicate population. Furthermore, where automation is impractical, and a team of researchers is employed to conduct the experiment, individual bias can skew observations, even in cases where subjective criteria are not directly used to measure the phenotypes. These problems are further compounded by the environmental variability that inconsistently affects phenotypic observation over both space and time. Unpredictable environmental conditions can also lead to a fair amount of missing data, which in turn will limit the statistical power to make inferences about the genotypic contribution to the phenotype. In addition to biological and environmental noise, variable assay quality can introduce further uncertainty and must be accounted for in any statistical models that are used to estimate parameters of interest.

Linear models have long been the mainstay of quantitative-genetic experiments, and are the most commonly applied statistical approach to understanding phenotypic variation. Traditionally, these models are fit using a variety of maximum-likelihood approaches (Lynch and Walsh 1997; Sorensen and Gianola 2002). These approaches are popular because they are fast and easy to use, and their long history has resulted in a wide availability of user-friendly software. However, maximum-likelihood methods have a number of serious limitations. Fundamentally, maximum-likelihood model fitting yields point estimates of parameters, ignoring the inherent uncertainty in their values. Parameters are then tested for statistical significance based on a threshold (typically the 5 % cut-off) and are excluded from further analysis if they are not “significant”. Finally, these statistical tests rely on restrictive assumptions about the distributions of model parameters. These constraints of maximum-likelihood model fitting affect experimental designs and data acquisition procedures, as well as biasing the resulting associations. More pointedly, these estimates perform well only when measurements are extensively replicated and normally distributed. Therefore, a great variety of procedures for data normalization and detection of outliers are necessary in order to meet the assumptions of the model. Unfortunately, these methods are often poorly motivated from a statistical point of view because they involve arbitrary thresholds for data exclusion. Despite these drawbacks, the speed and prevalence of maximum-likelihood methods make them useful as exploratory data analysis tools even in cases where the resulting estimates are not expected to be robust.

The Bayesian approach to statistical inference is fundamentally different and overcomes many of the limitations imposed by a maximum-likelihood approach. Instead of arriving at single most likely point estimates of parameters, the goal of Bayesian inference is to describe distributions of random variables of interest, taking into account uncertainty in all the other model parameters. This perspective on inference is thus much more in line with biological reality and should be preferable when dealing with phenotype data that have been contextualized by both the genotype and the environment. The drawback of Bayesian inference is its computational complexity. Historically, this complexity has been the disadvantage that held back widespread applications of Bayesian statistics. However, with the dramatic increase in computer power, it is now feasible to apply this approach to inference even when the data sets are large and multi-faceted. Furthermore, computer packages that make model building and analysis relatively simple and accessible to researchers without a programming background are starting to make an appearance (Lunn et al. 2009; Plummer 2003). Bayesian formulations of the standard quantitative-genetic models have been extensively studied (Sorensen and Gianola 2002), but these models can be computationally inefficient for large data sets. This is true for maximum-likelihood as well, but because Bayesian estimation involves the extra step of estimating full distributions rather than just point estimates of parameters the computational problems are particularly acute.

Appreciable improvements in computational stability and efficiency can be achieved by re-formulating the standard linear models in a hierarchical framework (Gelman et al. 2003; Gelman and Hill 2007). This framework is popular in the analyses of sociological data, and is now achieving more currency in genetics (Greenberg et al. 2010; Lenarcic et al. 2012). The basic idea is that quantitative-genetic experiments are inherently structured. For example, when an inbred line is evaluated in a number of environments, environmental effects can be nested within genotypic effects. Such nesting improves computational efficiency, increases power by incorporating data-driven pooling of observations from replicates (Gelman et al. 2003; Gelman and Hill 2007; Greenberg et al. 2010), and aids in biological interpretation of the results. Nesting environmental effects within genotypes has the added convenience of allowing the direct modeling of G×E interactions simply by estimating the regression slopes as they vary between inbred lines.

In cases where even modest numbers of outlier observations are present, Bayesian hierarchical models also out-perform similar maximum-likelihood approaches (Greenberg et al. 2010). Furthermore, it is straightforward to expand hierarchical models to include non-normal data (Gelman et al. 2003; Gelman and Hill 2007; Greenberg et al. 2010), handle unbalanced designs (Gelman et al. 2003; Greenberg et al. 2010), incorporate variable assay quality (Greenberg et al. 2010, 2011), account for outlier observations without using arbitrary thresholds to exclude them from the data (Greenberg et al. 2010, 2011; Lenarcic et al. 2012), and interrogate phenotypic networks by extending the analyses to multiple phenotypes through multivariate modeling (Greenberg et al. 2011). Finally, because the Bayesian approach integrates over the inherent uncertainty in a system and borrows power across the experiment through hierarchical modeling, it reduces the need for extensive biological replicates, and therefore maximizes the number of lines that can be evaluated in a given study (Greenberg et al. 2010).

That being said, while Bayesian hierarchical models are robust to many problems in experimental design and data acquisition, it is still a good idea to follow best practices when embarking on a quantitative-genetic experiment. Certain problems, such as putting all replicates for a line in a single block, lead to complete confounding of variables that cannot be resolved by any statistical treatment. Although it is possible to incorporate non-Gaussian data into Bayesian models, these extensions are typically computationally more expensive. For example, when modeling categorical data, one attempts to estimate an underlying continuous distribution that would yield the observed data when coerced to being discrete. Converting quantitative phenotypes (for example, the fraction of a plant tissue affected by disease) to an index (susceptibility class) leads to loss of information and an increase in model complexity. Likewise, summarizing replicate observations and reporting only means can lead to either increased noise when outliers are present or unwarranted precision. Such short cuts were defensible in the past, when computational power and storage capacity to handle large data sets was limited, but this is no longer the case and the data should be reported as “raw” as possible, and then modeled explicitly.

## Germplasm development and distributed phenotyping networks

Advances in phenotyping and genotyping technology, as well as data storage, and computational capacity are opening many new opportunities to extract meaningful inferences from even noisy biological data. New statistical models that account for biological uncertainty and estimate values of direct interest, rather than those dictated by computational convenience, promise to aid in the achievement of this goal. However, the value of any progress that may be gained through the marriage of next-generation phenotyping with modern genomic tools is predicated on the availability of diverse germplasm and genetically well-defined populations. Indeed associating genotype with phenotype in ways that address hypothesis-driven questions and empower crop improvement depends on the availability of appropriate germplasm resources to address specific questions.

The preservation of plant biodiversity in publicly available, international germplasm collections is of central importance to our quest to understand natural variation and to utilize that variation to meet the future needs of the planet. It is not unimaginable that we will be able to genomically characterize most of the accessions in the world’s repositories of genetic resources, but the sheer size of these collections, the broad range of adaptation they represent, import–export restrictions, and the genetic redundancy housed within their ranks presents a challenge for direct phenotypic evaluation. Targeted subsets of this variation need to be assembled so that available phenotyping resources can be efficiently used to evaluate them, taking advantage of economies of scale wherever possible (Glaszmann et al. 2010; McCouch et al. 2012). The development of shared populations with publically available, high-resolution genotype data will be critical for permitting the kind of distributed phenotyping necessary to understand genotype–phenotype relationships (Valdar et al. 2006). Examples of research communities that have developed these kinds of publicly shared germplasm resources include rice (Zhao et al. 2011a), maize (Yu et al. 2008), wheat (Neumann et al. 2011) *Arabidopsis* (Atwell et al. 2010), sorghum (Mitchell et al. 2008), barley (Pasam et al. 2012) and many other species (Zhu et al. 2008). The availability of these resources makes it possible for multiple researchers to interrogate the same genetic materials, phenotyping in environments and with technology and analytical expertise that are uniquely available to different research groups. Integrating such vast phenotypic datasets on common germplasm resources in well-structured databases will permit high-end analysis not just of the phenotypes themselves, but also of complex correlated phenotypic networks that represent a more accurate depiction of biological reality.

Additionally, more genetically structured resources such as chromosome segment substitution lines (Ali et al. 2010; Lu et al. 2011; Wang et al. 2012; Fukuoka et al. 2010; Xu et al. 2010; Zhang et al. 2011), multi-parent advanced generation inter-cross (MAGIC) populations (Huang et al. 2011, 2012; Rakshit et al. 2012), and nested association mapping (NAM) populations (Yu et al. 2008) will permit the interrogation of natural variation in elite genetic backgrounds that may be intractable otherwise. These genetically structured populations partition the variation in ways that facilitate the identification of exotic alleles that may have a significant impact on a phenotype of interest, but only when introgressed into the elite background. They also expedite the subsequent use of these resources as parents in a breeding program, helping expand the range of genetic variation available in an elite gene pool and opening up new opportunities to utilize natural variation to drive crop improvement.

## Conclusions

Ever since the first published QTL analysis (Sax 1923), genetics as a discipline has endeavored to shed light on the complexities of phenotypic variation. For most of recent memory, progress in understanding the genetic architecture of complex traits has been driven by improvement in genotyping technology. As a clear picture of the genome emerges, a renewed focus on understanding the nature of phenotypes will be necessary for continued advancement.

**Fig. 2:**
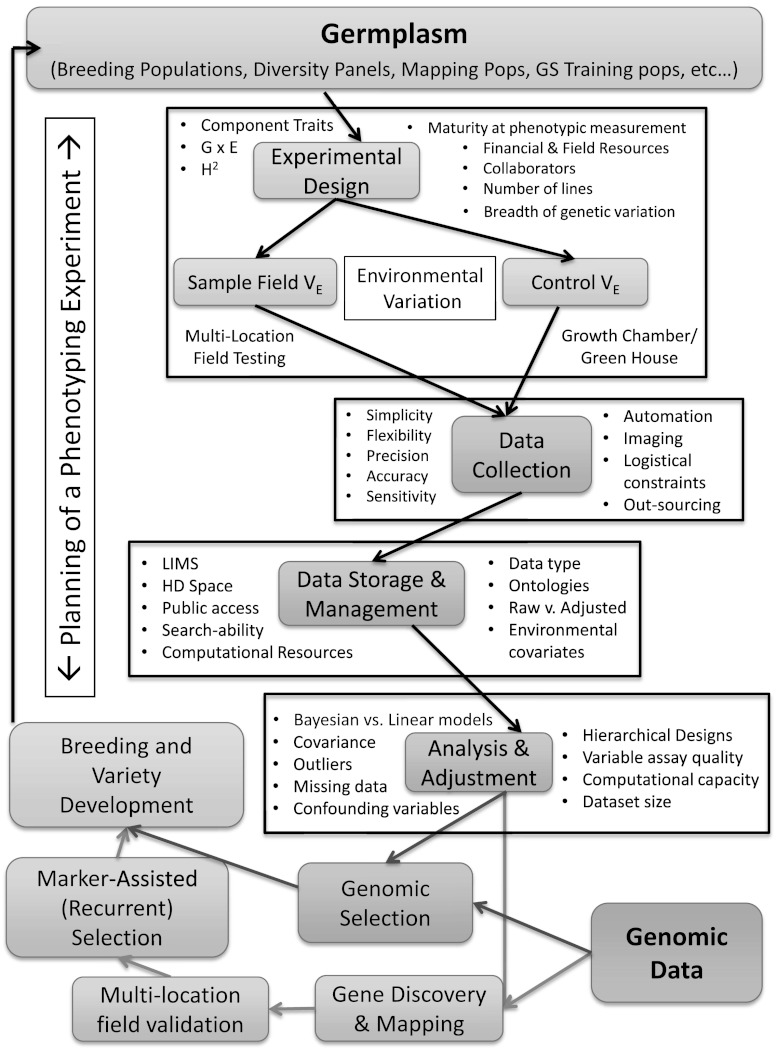
When combined with high-throughput genotyping on shared germplasm resources, and done in geographically distributed collaborative networks, next-generation phenotyping can empower both gene discovery and crop improvement. Central to that capacity is the careful and judicious use of modular technologies and managed environments. The use of standardized ontologies and Bayesian analysis then create a controlled vocabulary for describing the data and provide a way to integrate results across experiments by accounting for the unique signatures of biological noise generated by environmental covariates

We have discussed the role of phenotyping in gene discovery and crop improvement through both GWAS and GS, and we have attempted to understand the complexities incumbent on the association of genotype with phenotype under variable environmental conditions. We considered strategies that permit the collection of phenotypic data in quantitative ways as well as the development of modular technologies to accommodate the changing needs and opportunities of phenotyping in the future. We have pondered on the best practices for storing, cataloging, managing, and disseminating this information within a community, and suggested how this data might be combined with cutting edge statistical analysis to leverage increased computing capacity (Fig. 2). To conclude, we consider where some of the current deficiencies lie and highlight a few questions that still need answers.

Genotyping, while closing in on understanding the full extent of allelic variation in major crop species, is still years away from delivering on the quest to catalogue the world’s collection of DNA variants for an entire species. This requires assembly of multiple de novo reference genomes and re-sequencing of thousands of diverse lines to identify all of the SNPs, copy number variants, and other forms of DNA and epigenetic variation within a gene pool. As that information is generated, researchers will seek to annotate the functional significance of the SNPs and insertion/deletion polymorphisms, and design databases that can host this information and make it accessible and query-able for the research community. This is a real challenge because many functional variants do not fall within gene models, but are found as inter-genic regulatory elements or may condition gene expression through epigenetic pathways that contribute to quantitative phenotypic variation (Ding et al. 2012; Loehlin et al. 2010; Salvi et al. 2007; Zhou et al. 2012; Zhu and Deng 2012). This challenge also highlights the value of positional cloning to verify the functional nucleotide polymorphisms (FNP) rather than taking a candidate gene approach, as the FNP may not be found within a gene model at all. Additionally, for many years to come, the identification and characterization of rare alleles will remain a priority, despite the fact that both GWAS and GS have little power to detect their contributions to phenotypic variation.

Algorithms for optimizing signal-to-noise ratios in phenotypic experiments, pipelines for identifying GWAS peaks and extracting meaningful lists of candidate genes underlying those peaks are needed to help standardize association mapping studies. Functional annotation of QTL alleles and correspondence to the germplasm samples in which they are found would help link genetic research with breeding applications. Better tools for SNP haplotype visualization and management of high-volume SNP data need to be integrated into software platforms to facilitate the identification of functionally relevant SNPs that can be used for marker-assisted selection and as fixed variables in genomic prediction. As more and more phenotype data are collected and databased, tools to facilitate our understanding of intersecting phenotypic networks will shed light on the complex relationships within and between phenotypes (Yin and Struik 2008). This information will provide important insights about selection trade-offs and phenotypic correlations that are relevant to variety development and plant breeding.

Major questions about phenotypic variation, which we currently have limited capacity to answer, include: How does variation in regulatory elements manifest itself in the phenotype? Which environmental variables act as signals that regulate these genes and how do different allelic variants recognize those signals? What is the role of epistasis and epigenetics in determining phenotypic variation, or in phenotypic plasticity?

Approaching many of these questions will require more refined strategies of collecting and managing phenotype data. Many of the considerations that need to be addressed before making decisions about defining a phenotyping approach include: How easy is it to evaluate the phenotype? How quantitative is that measurement? Can the process be automated? If so, does it make economic sense to do so? What value would automation bring? What indirect factors will influence the phenotypic measurement? Can they be quantified? How much storage capacity do I need to maintain the raw or processed phenotypic data? How will the data be organized so that it is both query-able and understandable? What data processing needs must be considered before the phenotype is biologically meaningful? Do I have the skills in-house or appropriate collaborators in place to realize a sophisticated analysis of the data? Answers to these questions will depend entirely on the purpose and intention of collecting phenotypic data to start with, and of course the nature of the phenotype itself.

The phenotype of an organism is fundamentally a manifestation of a genotype’s interaction with the environment. With increased allocation of funding and intellectual investment over the next decade, advances in phenotyping will enhance our ability to associate that data with the genotypic and environmental variables to simultaneously and synergistically drive gene discovery efforts aimed at understanding the genetic basis of quantitative phenotypic variation and fuel the development of genomic prediction models for crop improvement. As these two drivers of genetic analysis feed into each other, not only will tremendous gains be made in comprehending the biology of plants, but we will also ensure continued advancement in crop improvement aimed at meeting the demands of a growing population.
